# The Natural Coagulation Inhibitors, Inflammation, and Thrombotic Risk in Sickle Cell Patients in Cameroon: An Analytical Cross‐Sectional Study

**DOI:** 10.1155/ah/1400762

**Published:** 2026-05-18

**Authors:** Romaric De Manfouo Tuono, Josué Louokdom Simo, Paule Rita Ngamgwe Tchouanmo, Ingrid Worti Sulem Yong, Maryline Seuko Njopwouo, Claude Tagny Tayou

**Affiliations:** ^1^ Higher Institute of Health Sciences, Université Des Montagnes, Bangangté, Cameroon, udesmontagnes.org; ^2^ Faculty of Medicine and Biomedical Sciences, Université De Yaoundé 1, Yaoundé, Cameroon, uninet.cm

**Keywords:** antithrombin, inflammation, protein C, protein S, sickle cell disease, thrombotic risk

## Abstract

**Introduction:**

Thrombotic risk refers to the likelihood of a thrombus, or blood clot, forming in the vascular system, which can obstruct blood flow and cause serious complications. This risk is closely linked to the function of physiological coagulation inhibitors. The causes of thrombosis are multiple, including damage to the vascular wall caused by blood vessel injury, trauma, surgery, or vascular inflammation; these can initiate stasis, or slow blood flow, thus promoting blood cell aggregation and clot formation. These factors are encountered during several pathological processes, including sickle cell disease. This study aimed to evaluate physiological coagulation inhibitors and their association with inflammation and to deduce the thrombotic risk in patients with sickle cell disease.

**Materials and methods:**

We conducted an analytical cross‐sectional study over 1 year. The Bafoussam Regional Hospital served as the framework for this research. The study population consisted of homozygous sickle cell patients regularly followed in the hospital and recruited in the hematology department. The collected blood samples were sent to the Hematology and Hemostasis laboratory. The analyses carried out were the hemogram by the flow cytometry method; the determination of the activity of protein C, protein S, and antithrombin with a coagulometer by the magnetic method; and finally, the exploration of inflammation from CRP dosage by the ultrasensitive method and the dosage of interleukin‐6 by the ELISA sandwich method. The data obtained were recorded in an Excel 2013 spreadsheet and analyzed using the statistical software *R* Version 4.1.1.

**Results:**

A total of 150 homozygous sickle cell patients were included in this study. Of these, 98 (65.34%) presented a thrombotic risk. A high frequency of anemia (94.7%) was reported. Moreover, 82.7% and 64.0%, respectively, of leukocytosis and thrombocytosis were reported. An elevation of inflammatory parameters was observed characterized by an elevation of CRP (100% of patients at risk versus none in the group without it) and a significantly higher median IL‐6 level in the group of patients at thrombotic risk (50.1 [28.7–76.6] [pg/mL]) compared to those without it (32.3 [14.1–44.4] pg/mL) (*p* = 0.001). A decrease in coagulation inhibitors was observed, notably a significant decrease in protein C in patients with thrombotic risk compared to those without, with respective proportions of 71.6% and 50% (*p* = 0.001). The median antithrombin level was 79.0% (77.0–92.0) in patients with thrombotic risk and 94.0% (84.0–102) in those without (*p* < 0.001). Patients at risk of thrombosis had a significant decrease in protein S (76.6% vs. 50% [*p* < 0.001]); the decrease in the latter was identified as a predictive factor of thrombotic risk during sickle cell disease (aOR = 1.5 [1.1; 5.2]; *p* = 0.04).

**Conclusion:**

This study reports a significant frequency of thrombotic risk in the sickle cell population characterized by a decrease in physiological coagulation inhibitors. This highlights the need for a systematic exploration of these markers for the prevention of cardiovascular disease in this patient group.

## 1. Introduction

Thrombotic risk refers to the likelihood of a thrombus, or blood clot, forming in the vascular system, which can obstruct blood flow and cause serious complications, such as deep vein thrombosis (DVT) or pulmonary embolism (PE) [1]. There are many causes of thrombosis, including damage to the vascular wall caused by injuries to blood vessels caused by trauma, surgery, or vascular inflammation [1, 2]; these can initiate the formation of stasis, or the slowing of blood flow, thus promoting the aggregation of blood cells and the formation of clots [3, 4]. Also, abnormalities in clotting factors can make blood more likely to form clots [5, 6]. This thrombotic risk is closely linked to the function of physiological coagulation inhibitors and essential proteins that regulate the balance between the formation and dissolution of blood clots [7, 8]. The most important physiological inhibitors include protein C, protein S, and antithrombin. Together, they control the activity of procoagulant factors and prevent excessive formation of thrombin, the key enzyme in the coagulation cascade [9]. Protein C is activated by thrombin in the presence of thrombomodulin (a receptor on the vessel wall). Once activated, with the help of its cofactor, inactive protein S, it reduces thrombin production, thereby decreasing the formation of fibrin (the protein network that forms the basis of clots). When protein C or protein S levels are low, inactivation of factors Va and VIIIa is insufficient, leading to increased thrombin production. This creates a hypercoagulable state, where the risk of abnormal clot formation increases, potentially leading to thrombosis [10, 11]. Furthermore, antithrombin is a protein that neutralizes several coagulation factors, particularly thrombin and factor Xa [6, 12]. By acting as a potent thrombin inhibitor, antithrombin prevents excessive clot formation. Antithrombin deficiency (whether hereditary or acquired) reduces the body’s ability to regulate clotting, significantly increasing the risk of thrombosis. Patients with low antithrombin levels are more likely to develop DVT and other thromboembolic complications [13, 14]. The above is encountered during certain pathological processes, including sickle cell disease (SCD).

In fact, SCD is a hereditary blood disorder characterized by the production of abnormal hemoglobin molecules that give red blood cells a crescent or sickle shape [15]. This morphological alteration makes red blood cells more rigid and less able to pass through small blood vessels, leading to vaso‐occlusive crises, tissue damage, and chronic inflammation [16]. In addition to the well‐known clinical manifestations, SCD is characterized by a state of hypercoagulability due to decreases in anticoagulant proteins of the hemostatic system [17]. These complications increase the risk of thrombosis in patients with SCD, leading to increased morbidity and risk of serious complications, such as DVT, PE, and stroke [18, 19]. The high thrombotic risk in SCD is linked to several pathophysiological factors. Sickle red blood cells aggregate more easily in microvessels, causing damage to the vascular wall and triggering excessive activation of coagulation. SCD is also associated with systemic inflammation and oxidative stress, both of which contribute to chronic activation of endothelial cells and platelets, thus increasing the tendency to form clots [20–22]. This process is accentuated by the reduction of physiological coagulation inhibitors, such as antithrombin and proteins C and S, which are often deficient in sickle cell patients, thus reinforcing the imbalance between procoagulant and anticoagulant factors [23–25].

This thrombotic risk in sickle cell patients poses a therapeutic challenge, as anticoagulant treatments must be tailored to effectively reduce clot formation while preventing excessive bleeding. Recognizing and managing these thrombotic risks is essential to improve the quality of life and reduce serious complications in patients with SCD. However, despite the high prevalence of these complications, the exact mechanisms of thrombosis in sickle cell patients remain an area of active research and require a better understanding to optimize prevention and treatment strategies. With this in mind, we set out to evaluate physiological coagulation inhibitors and the thrombotic risk in homozygous sickle cell patients with inflammatory conditions to prevent these complications, improve patient care, and provide further insight into the subject in the Cameroonian context.

## 2. Methods

### 2.1. Ethical Considerations

This study was conducted after obtaining authorization for collection from the Bafoussam Regional Hospital (No. 656/L/MINSANTE/SG/DRSPO/HRB/D). The University of the Mountains issued an ethical clearance (No. 2024/162/UdM/PR/CEAQ). Patients were informed about the purpose of the study and the benefits and risks of participating. The information obtained from each participant was kept confidential. This study was conducted in strict compliance with the principles of medical research as set out in the Declaration of Helsinki [26].

### 2.2. Study Design and Subjects

The study was of a cross‐sectional analytical type and conducted over 1 year in 2024. The study population consisted of homozygous sickle cell patients who came for routine consultations and were regularly monitored at the Hematology Department of the Bafoussam Regional Hospital. These patients were in the defined stationary phase for patients whose last VOC dates back more than 6 months.

An estimate of the size of the population to be included was made from the arithmetic formula defined by Lorentz, taking into account the frequency of SCD in Cameroon (2%–3%) and the frequency of predisposition to thrombotic risk during SCD defined by Chekkal et al. [27] who reported 63% and having defined the thrombotic risk from significant decrease in levels of proteins C and S in patients with sickle cell homozygous compared to controls. Sociodemographic, anthropometric, and clinical data were collected from the participants.

The selection criteria for participants were as follows: All SS patients of both sexes who consented to participate in the study and provided the required information were consecutively included, nonexhaustively. Patients with inflammatory conditions, such as infections and those on anticoagulant therapy, were excluded, as were those who had received a blood transfusion within the last 3 months; pregnant women and those taking hormonal contraceptive pills were also excluded. Also, patients who had liver failure with increased prothrombin time (PT) and/or clinical signs of cirrhosis and those who had kidney failure or cancer were excluded. A total of 150 patients were included.

### 2.3. Data Collection Tools and Methods

A volume of 4 mL of blood was collected from participants in EDTA, 3.2% citrate, and dry tubes, respectively, for hematological, hemostasis, and inflammation analyses; each tube was sent to the appropriate laboratory.

Hematological parameters were analyzed using a HumaCount 30TS hematology automaton. Blood counts were performed along with blood smears for differential leukocyte count and reticulocyte counts according to the protocols described by Rovanowsky. The slides obtained were read under an IRMECO binocular microscope, and the results were interpreted according to WHO recommendations.

The phenotype of each participant was confirmed by performing hemoglobin electrophoresis on agarose gel. The final products were stained with Ponceau red and visualized using a densitometer. The exploration of natural anticoagulants of hemostasis was carried out from the dosage of protein C, protein S, and antithrombin on the SOCIMED coagulometer using a magnetic principle.

Thus, protein C activity was determined using the “HEMOCLOT” kits (*ref. CK031K, 155, Eragny Street, France*) which use a coagulant method, using the activated partial thromboplastin time (aPTT), in the presence of protein C activator (PCA) (Protac, extracted from the venom of the snake Agkistrodon Contortrix), phospholipids, contact phase activator, and calcium. The principle of the test used is as follows: The diluted patient plasma to be tested is first mixed with the PC‐deficient plasma (R1). In a second step, the activating reagent (R2), in a constant and optimized concentration, is added. Coagulation is triggered by the addition of calcium (Ca^2+^). The clotting time is measured. As protein C is the limiting factor, a direct linear relationship, in biologarrhythmic coordinates, results between the protein C concentration and the corresponding clotting time.

Protein S activity was also determined using the “HEMOCLOT” kits (*BIOPHEN, 155, Eragny Street, France*), however, using the aPTT, triggered by factor IXa in the presence of phospholipids, calcium, and a constant and excess amount of PCA. The principle of the test used is as follows: The diluted plasma to be tested is mixed with PS‐deficient plasma (R1). The activating reagent (R2), at a constant and optimized concentration, is added. Coagulation is triggered by the addition of calcium (Ca^2+^). As PS is the limiting factor, a direct relationship results between PS concentration and the corresponding measured clotting time.

Finally, antithrombin activity was determined according to the “HEMOCLOT” kits (*ref. CK002K155, Eragny Street, France*) whose principle is as follows: For measuring dabigatran or any other direct thrombin inhibitors (DTIs) in plasma, first, the diluted tested plasma is mixed with normal pooled human plasma (R1). Clotting is then initiated by adding a constant amount of highly purified human thrombin, essentially in the *α*‐form (R2). The clotting time measured is directly related to the concentration of dabigatran or assayed DTI in the tested plasma.

The reference values of the activities of each of these proteins used were 70%–140%, 60%–140%, and 80%–120% for protein C, protein S, and antithrombin, respectively. Thus, thrombotic risk was defined as a decrease of the activity in at least protein C or antithrombin, or these combined with a decrease in protein S, as suggested by these authors [27–29].

Inflammation was assessed using hypersensitive CRP (hs‐CRP) assays using the specific PA54 protein analyzer and Genrui brand reagent kits (*Lotus NL B.V., The Hague, Netherlands*). hs‐CRP was used as a diagnostic marker of inflammation as defined by previous authors [30, 31]. Also, the concentration of interleukin‐6 was determined according to the kits “Elabscience R” kits (*Elabscience Biotech Inc., United States*), whose principle is based on ELISA sandwich. The analyses were carried out on an ELISA chain (*ELx50 TM Automated Strip Washer brand BIOTEK*). Inflammation was determined from quantitative methods and defined for hs‐CRP cut‐off values > 6 mg/L [31], associated with IL‐6 > 5 pg/mL (as defined by the test kits). This made it possible to define patients with inflammation and those without.

### 2.4. Data Management and Statistical Analysis

The collected data were recorded in an Excel 2016 spreadsheet and analyzed using the *R* statistical software Version 4.1.1. The Smirnov–Kolmogorov test was used to determine the normal distribution or not of the different data. Quantitative variables were grouped into “low,” “normal,” or “high” according to the reference values defined by the kits of the different reagents and the WHO. Thus, the median and the interquartile range were used to present the quantitative variables, and the qualitative variables were presented in the form of frequency. The Fisher exact test was used to compare the frequencies, and the Wilcoxon test was used to compare the medians. Logistic regression analyses based on univariate and multivariate models were used to identify the factors associated with thrombotic risk during this study. Spearman’s correlation coefficient (*r*) was used to determine the correlation between IL‐6 levels and the measured thrombotic markers. The significance threshold was set at *p* < 0.05.

## 3. Results

### 3.1. Distribution of the Study Population According to Sociodemographic, Anthropometric, and Clinicobiological Characteristics

#### 3.1.1. Sociodemographic and Anthropometric Characteristics of the Population

The number of sickle cell patients included in this study was 150. Of these, 98 (65.34%) presented a thrombotic risk. Table 1 describes the distribution of the study population according to sociodemographic and anthropometric characteristics of thrombotic risk.

**TABLE 1:** Sociodemographic and anthropometric characteristics of the population according to thrombotic risk.

Parameters		Thrombotic risk–negative *N* = 52	Thrombotic risk–positive *N* = 98	Total *N* = 150	*p*‐value
Sex:	Feminine	26 (50.0)	42 (42.9)	68 (45.3)	0.507
	Masculine	26 (50.0)	56 (57.1)	82 (54.7)	

Marital status	Single	50 (96.2)	96 (98.0)	146 (97.3)	0.610
	Married	2 (3.85)	2 (2.04)	4 (2.67)	

School level	Not in school	2 (3.85)	0 (0.00)	2 (1.33)	0.175
	Primary	44 (80.79)	90 (91.84)	134 (89,33)	
	Secondary	4 (7.69)	6 (6.12)	10 (6,67)	
	University	4 (7.69)	2 (2.04)	6 (4)	
	Age, Med[q1‐q3]	10.0 (5.00–17.0)	9.00 (4.00–14.0)	9.00 (4.25–14.0)	0.641
	BMI, Moy ± sd	17.1 ± 3.06	17.6 ± 3.17	17.4 ± 3.13	0.346

BMI:	Underweight	38 (73.1)	56 (57.1)	94 (62.7)	0.081
	Normal	14 (26.9)	42 (42.9)	56 (37.3)	

Abbreviation: BMI: body mass index.

Table 1 reports a male predominance (54.7%) for a sex ratio of 1.2. The median age of the included population is 9.00 (4.25–14.0) years. Of the patients included, 94 (62.7%) were underweight, although these parameters do not differentiate according to thrombotic risk.

#### 3.1.2. Clinical Characteristics, History, and Lifestyle of the Population

Table 2 describes the distribution of the study population according to the characteristics, clinical history, and lifestyle habits of the population, according to the thrombotic risk.

**TABLE 2:** Clinical characteristics, history, and lifestyle habits of the population.

Parameters		Thrombotic risk–negative *N* = 52	Thrombotic risk–positive *N* = 98	Total *N* = 150	*p*‐value
Knowledge about sickle cell disease:	No	8 (15.4)	20 (20.4)	28 (18.7)	0.595
	Yes	44 (84.6)	78 (79.6)	122 (81.3)	

Sickle cell disease in the family:	No	22 (42.3)	60 (61.2)	82 (54.7)	0.041
	Yes	30 (57.7)	38 (38.8)	68 (45.3)	

History of infectious attacks:	No	32 (61.5)	76 (77.6)	108 (72.0)	0.059
	Yes	20 (38.5)	22 (22.4)	42 (28.0)	

History of anemic crises:	No	30 (57.7)	44 (44.9)	74 (49.3)	0.187
	Yes	22 (42.3)	54 (55.1)	76 (50.7)	

History of vaso‐occlusive crises:	No	24 (46.2)	52 (53.1)	76 (50.7)	0.526
	Yes	28 (53.8)	46 (46.9)	74 (49.3)	

VOC frequency/month:	1.00 (0.00–1.00)	1.00 (0.00–2.00)	1.00 (0.00–2.00)	0.719	

Iron treatment:	No	32 (61.5)	48 (49.0)	80 (53.3)	0.195
	Yes	20 (38.5)	50 (51.0)	70 (46.7)	

Folic acid treatment:	52 (100)	98 (100)	150 (100)	.	

Hydroxyurea:	No	42 (80.8)	96 (98.0)	138 (92.0)	< 0.001
	Yes	10 (19.2)	2 (2.04)	12 (8.00)	

History CD et CD:	No	52 (100)	94 (95.9)	146 (97.3)	0.299
	Yes	0 (0.00)	4 (4.08)	4 (2.67)	

*Note:* CD =  cerebrovascular accident.

Abbreviations: CD = cardiovascular diseases; VOC: vaso‐occlusive crisis.

From Table 2, the included population knew about the disease, and 68 (45.3) of the included patients had sick siblings. The included patients had a history of anemic crises, vaso‐occlusive crises, and infectious crises, although this did not differentiate according to thrombotic risk. The included patients were under iron treatment (46.7%), folic acid treatment (100%), and hydroxyurea (HU) treatment (8%).

### 3.2. Distribution of the Study Population According to the Blood Count Parameters

#### 3.2.1. Parameters and Disorders of the Blood Count in the Population According to the Red Line

Table 3 shows the profile and frequency of blood count disorders in the population according to the red lineage as a function of thrombotic risk.

**TABLE 3:** Blood count parameters and disorders in the population according to the red lineage as a function of thrombotic risk.

Parameters		Thrombotic risk–negative *N* = 52	Thrombotic risk–positive *N* = 98	Total *N* = 150	*p*‐value
RBC, Med[q1‐q3] (T/L)		2.62 (2.11–3.01)	2.72 (2.48–3.12)	2.70 (2.35–3.09)	0.022^b^ ^,^ ^∗^
RBC:	Low	52 (100)	86 (87.8)	138 (92.0)	0.011^∗^
	High	0 (0.00)	2 (2.04)	2 (1.33)	
	Normal	0 (0.00)	10 (10.2)	10 (6.67)	
HB, Med[q1‐q3] (g/dL)		7.55 (6.50–8.10)	7.80 (7.10–8.60)	7.60 (7.00–8.28)	0.089^a^
HB:	Low	52 (100)	90 (91.8)	142 (94.7)	0.051^a^
	Normal	0 (0.00)	8 (8.16)	8 (5.33)	
Degree of anemia	Mild	16 (30.8)	30 (30.6)	46 (30.7)	0.169^a^
	Moderate	30 (57.7)	52 (53.1)	82 (54.7)	
	Normal	0 (0.00)	8 (8.16)	8 (5.33)	
	Severe	6 (11.5)	8 (8.16)	14 (9.33)	
HTE, Med[q1‐q3] (%)		21.7 (18.1–22.7)	22.1 (20.3–24.6)	21.8 (19.8–23.5)	0.042^b^ ^,^ ^∗^
HTE:	Low	52 (100)	90 (91.8)	142 (94.7)	0.095^a^
	High	0 (0.00)	2 (2.04)	2 (1.33)	
	Normal	0 (0.00)	6 (6.12)	6 (4.00)	
MCV, Med[q1‐q3] (fL)		82.0 (77.4–86.0)	80.0 (74.0–87.0)	81.0 (74.2–86.0)	0.384^b^
MCV:	Low	14 (26.9)	46 (46.9)	60 (40.0)	0.027^∗^
	Normal	38 (73.1)	52 (53.1)	90 (60.0)	
MCH, Med[q1‐q3] (pg/*c*)		29.2 (26.8–31.1)	28.3 (25.0–31.2)	28.8 (25.6–31.2)	0.107^a^
MCH:	Low	14 (26.9)	38 (38.8)	52 (34.7)	0.204^a^
	Normal	38 (73.1)	60 (61.2)	98 (65.3)	
MCHC, Med[q1‐q3] (g/L)		35.7 (35.3–37.4)	35.2 (33.6–36.3)	35.6 (34.3–36.7)	0.008^b^ ^,^ ^∗^
MCHC:	Low	2 (3.85)	8 (8.16)	10 (6.67)	0.495^a^
	Normal	50 (96.2)	90 (91.8)	140 (93.3)	
Reticulocytes, Med[q1‐q3] (G/L)		357,5 (300–377)	355 (275–377)	355 (295,7–377)	0.669^b^
Reticulocytes:	High	52 (100)	90 (91.8)	142 (94.7)	0.051^a^
	Normal	0 (0.00)	8 (8.16)	8 (5.33)	

*Note:* Med [q1‐q3] = median and interquartile range; MCH = mean corpuscular hemoglobin content; M: mean; RDB = red blood cell.

Abbreviations: G/L = giga/liter, MCHC = mean corpuscular hemoglobin concentration, MCV = mean corpuscular volume, Sd = standard deviation, T/L = tera/liter.

^∗^significant difference at *p* < 0.05.

^a^Fisher’s test.

^b^Wilcoxon test.

The red blood cell hematological parameters described in Table 3 above report a high frequency of anemias (94.7%), although they do not differentiate according to thrombotic risk. Of the anemias reported, 40.0% were microcytic, 34.7% hypochromic, and 94.7% regenerative, although these parameters do not differentiate according to thrombotic risk.

#### 3.2.2. Parameters and Disorders of the Blood Count in the Population According to the White and Platelet Lineage

Table 4 shows the profile and frequency of blood count disorders in the population according to the white and platelet lineage as a function of thrombotic risk.

**TABLE 4:** Parameters and disorders of the blood count in the population according to the white and platelet lineage as a function of thrombotic risk.

Parameters		Thrombotic risk–negative N = 52	Thrombotic risk–positive N = 98	Total N = 150	*p*‐value
WBC, Med[q1‐q3] (G/L)		15.6 (11–20.1)	15.7 (12.6–21.3)	15.7 (12.1–20.4)	0.580*^b^*

WBC:	High	44 (84.6)	80 (81.6)	124 (82.7)	0.816*^a^*
	Normal	8 (15.4)	18 (18.4)	26 (17.3)	

Lymphocytes, Med[q1‐q3] (G/L)		5.7 (4.3–9.4)	6.4 (4.1–8.8)	6.01 (4,3–8,9)	0.981*^b^*

Lymphocytes:	High	42 (80.8)	76 (77.6)	118 (78.7)	0.804*^a^*
	Normal	10 (19.2)	22 (22.4)	32 (21.3)	

Monocytes, Med[q1‐q3] (G/L)		1.8 (1.3–3.2)	2.2 (1.8–3.01)	2.1 (1.5–3.01)	0.029^b^ ^,^ ^∗^

Monocytes:	High	46 (88.5)	98 (100)	144 (96.0)	0.001^∗^
	Normal	6 (11.5)	0 (0.00)	6 (4.00)	

Granulocytes, Med[q1‐q3] (G/L)		6.04 (4.2–8.5)	6.6 (4.8–8.8)	6.2 (4.7–8.6)	0.236*^b^*

Granulocytes:	Low	0 (0.00)	2 (2.04)	2 (1.33)	0.134*^a^*
	High	14 (26.9)	40 (40.8)	54 (36.0)	
	Normal	38 (73.1)	56 (57.1)	94 (62.7)	

Platelets, Med[q1‐q3] (G/L)		488 (296–597)	471 (328–597)	481 (310–597)	0.931

Platelets	Low	4 (7.69)	2 (2.04)	6 (4.00)	0.079
	High	36 (69.2)	60 (61.2)	96 (64.0)	
	Normal	12 (23.1)	36 (36.7)	48 (32.0)	

*Note:* Med [q1‐q3] = median and interquartile range.

Abbreviations: G/L = giga/liter, T/L = tera/liter, WBC = white blood cells.

^∗^significant difference at *p* < 0.05.

^b^Wilcoxon test.

^a^Fisher’s test.

Table 4 reports a significant frequency of leukocytosis (82.7%) in the study population, although not differentiated according to thrombotic risk. Moreover, 96 (64.0%) patients presented cases of thrombocytosis.

### 3.3. Distribution of the Study Population According to Physiological Coagulation Inhibitors and Inflammatory Parameters

Table 5 presents physiological coagulation inhibitors and inflammatory parameters according to thrombotic risk.

**TABLE 5:** Physiological inhibitors of coagulation and inflammatory parameters in the population as a function of thrombotic risk.

Parameters		Thrombotic risk–negative *N* = 52	Thrombotic risk–positive *N* = 98	Total *N* = 150	*p* *-*value
Protein C (%), Med[q1‐q3]		76.0 (67.0–83.0)	64.0 (51.0–90.0)	71.0 (53.2–83.8)	0.012^b^, ^∗^
Protein C:	Low	14 (26.9)	56 (57.1)	70 (46.7)	0.001^a^ ^∗^
	Normal	38 (73.1)	42 (42.9)	80 (53.3)	
Protein S (%), Med[q1‐q3]		56.5 ± 7.77	54.6 ± 6.37	55.2 ± 6.93	0.124
Protein S:	Low	26 (50.0)	76 (77.6)	102 (68.0)	0.001^a^ ^∗^
	Normal	26 (50.0)	22 (22.4)	48 (32.0)	
Antithrombin (%), Med[q1‐q3]		94.0 (84.0–102)	79.0 (77.0–92.0)	84.0 (78.0–97.8)	< 0.001^b^ ^,^ ^∗^
Antithrombin:	Low	0 (0.00)	56 (57.1)	56 (37.3)	< 0.001^a,^ ^∗^
	Normal	52 (100)	42 (42.9)	94 (62.7)	
CRP (mg/L), Med[q1‐q3]		4.50 (3.00–4.93)	11.5 (8.72–22.5)	8.67 (4.93–14.7)	< 0.001^b^ ^,^ ^∗^
CRP:	High	0 (0.00)	98 (100)	98 (65.3)	< 0.001^a,^ ^∗^
	Normal	52 (100)	0 (0.00)	52 (34.7)	
Inflammation:	No	52 (100)	0 (0.00)	52 (34.7)	< 0.001^a,^ ^∗^
	Yes	0 (0.00)	98 (100)	98 (65.3)	
IL‐6 (pg/mL), Med[q1‐q3]		32.3 (14.1–44.4)	50.1 (28.7–76.6)	42.0 (19.9–73.3)	< 0.001^b^ ^,^ ^∗^
IL‐6:	High	52 (100)	94 (95.9)	146 (97.3)	0.299
	Normal	0 (0.00)	4 (4.08)	4 (2.67)	

*Note:* Med [q1‐q3] = median and interquartile range; IL‐6 = interleukin‐6.

^∗^significant difference at *p* < 0.05.

^a^Fisher’s test.

^b^Wilcoxon test.

The general population presented a decrease in protein C characterized by a significant decrease in the latter in patients with thrombotic risk versus those without, with respective proportions of 71.6% and 50% (*p* = 0.001). Moreover, all patients with thrombotic risk had a decrease in antithrombin with a significantly low median level (79.0% [77.0–92.0]) compared to those without risk (94.0% [84.0–102]) (*p* < 0.001). Also, patients with thrombotic risk presented a significant decrease in protein S (76.6% vs. 50%, *p* < 0.001). Furthermore, patients with thrombotic risk had an elevation of inflammatory parameters characterized by an elevation of CRP (100% of patients with the risk vs. none in the group without it) and a significantly higher median IL‐6 level in the group of patients with thrombotic risk (50.1 [28.7–76.6] [pg/mL]) compared to those without it (32.3 [14.1–44.4] pg/mL) (*p* = 0.001).

### 3.4. Factors Associated With Thrombotic Risk in the Population: Univariate and Multivariate Analyses

Table 6 describes the factors associated with thrombotic risks in the population during this study according to the univariate and multivariate models.

**TABLE 6:** Factors associated with thrombotic risk in the population according to the univariate and multivariate models.

Parameters		Thrombotic risk–negative *N* = 52	Thrombotic risk–positive *N* = 98	Univariate analysis OR (95% CI)	*p*‐value	Multivariate analysis: aOR (95% CI)	*p*‐value
Sex	Feminine	26 (50.0)	42 (42.9)	0.75 (0.38; 1.47)	0.410	/	/
	Masculine	26 (50.0)	56 (57.1)	Ref.	Ref.	/	/

BMI	Thin	38 (73.1)	56 (57.1)	0.49 (0.24; 1.02)	0.057	/	/
	Normal	14 (26.9)	42 (42.9)	Ref.	Ref.	/	/

Hydroxyurea	No	42 (80.8)	96 (98.0)	11.4 (2.40; 54.4)	0.001^∗^	8.00 (1.26–68.85)	0.034^∗^
	Yes	10 (19.2)	2 (2.04)	Ref.	Ref.	Ref	Ref

HB	Low	52 (100)	90 (91.8%)	0.00 (0.00.)	Ref.	/	/
	Normal	0 (0.00%)	8 (8.16%)	Ref.	0.030^∗^	/	/

Prot C	Low	14 (26.9)	56 (57.1)	3.62 (1.74; 7.52)	< 0.001^∗^	/	/
	Normal	38 (73.1)	42 (42.9)	Ref	Ref.	/	/

Prot S	Low	26 (50.0)	76 (77.6)	3.45 (1.68; 7.11)	0.001^∗^	1,5 (1.1; 5.2)	0.04^∗^
	Normal	26 (50.0)	22 (22.4)	Ref.	Ref.	ref	ref

Antithrombin	Low	0 (0.00)	56 (57.1)	. (.;.)	< 0.001^∗^	/	/
	Normal	52 (100)	42 (42.9)	Ref.	Ref.	/	/

CRP	High	0 (0.00)	98 (100)	(.;.)	0.000^∗^	/	/
	Normal	52 (100)	0 (0.00)	Ref.	Ref.	/	/

IL‐6	High	52 (100)	94 (95.9)	0.00 (0.00.)	0.178	/	/
	Normal	0 (0.00)	4 (4.08)	Ref.	Ref.	/	/

MCV	Low	14 (26.9)	46 (46.9)	2.40 (1.16; 4.98)	0.018^∗^	1.00 (0.25–4.44)	1.000
	Normal	38 (73.1)	52 (53.1)	Ref.	Ref.	/	/

*Note:* multivariate analysis; , IL‐6 = interleukin‐6.

Abbreviations: aOR = adjusted odds ratio, 95% CI = 95% confidence interval, OR = odds ratio.

^∗^significant difference at *p* < 0.05.

From Table 6, according to the univariate model, decreases in protein C, protein S, and nonuse of HU treatment constitute predictive factors of thrombotic risk with OR = 3.62 ([1.74; 7.52]; *p* < 0.001), OR = 3.45 ([1.68; 7.11]; *p* < 0.001), and OR = 11.4 ([2.40; 54.4]; *p* < 0.001), respectively. Moreover, according to the multivariate model, the decrease in protein S and the absence of HU treatment are identified as predictive factors of thrombotic risk with aOR = 1.5 ([1.1; 5.2]; *p* = 0.04) and aOR = 8.00 ([1.26–68.85]; *p* = 0.034), respectively.

### 3.5. Correlation Analysis Between IL‐6 Levels and the Measured Thrombotic Markers

Table 7 describes the correlation analysis between IL‐6 levels and the measured thrombotic markers.

**TABLE 7:** Correlation analysis between IL‐6 levels and the measured thrombotic markers.

Parameters	IL‐6	
	r	*p*‐value
Protein C	−0.16	0.051
Protein S	0.034	0.68
Antithrombin	0.04	0.6

*Note:* r =  Spearman’s correlation coefficient.

From Table 7, we report a positive correlation between IL‐6 and protein S as well as antithrombin, although it was not statistically significant (*r* = 0.034, *p* = 0.68 and *r* = 0.04, *p* = 0.6, respectively). Furthermore, a negative correlation between IL‐6 and protein C was observed, although it was not statistically significant (*r* = −0.16; *p* = 0.051).

## 4. Discussion

The purpose of this study was to evaluate physiological coagulation inhibitors and their association with inflammation, and to deduce the thrombotic risk in sickle cell patients. Indeed, inflammation and abnormalities of hemostasis in general, and in particular those of physiological coagulation inhibitors, constitute a vicious circle and a heavy burden in sickle cell patients, predisposing them to a high thrombotic risk [22, 32, 33]. Patients with SCD are exposed to multiple factors that promote inflammation [34], thrombin generation, and coagulation imbalance, including increased plasma levels of thrombin markers, decreased natural anticoagulant proteins, abnormal fibrinolytic system activation, and enhanced tissue factor expression during crisis states. These factors collectively contribute to a heightened risk of thromboembolic events [19, 35, 36]. From this evaluation during this study, it appears that 98 (65.34%) patients presented a thrombotic risk characterized by the affection of the homeostasis of the above‐mentioned parameters. This would be the result of intrinsic and extrinsic factors to the disease characterized by vaso‐occlusive crises, infections, and anemic crises; the irregular blood flow which results from it would favor frequent activation of coagulation; and this repeated activation favors the formation of thrombin and fibrin, which ultimately increases the risk of clot formation [37, 38]. These results are similar to those of Quari et al. [28] who reported a 71% thrombotic risk during their study and also those of Mohamed Chekkal et al. [27] who reported 63%.

This study reports a male predominance (54.7%) for a sex ratio of 1.2. The median age of the included population was 9.00 (4.25–14.0) years, corresponding to a relatively young age, which could be explained by the severe and chronic complications, such as heart failure, pulmonary hypertension, and common infections in young adults with SCD, contributing to the reduction in overall life expectancy [39]. These results are also close to the observations made by several authors [40–43]. Of the patients included, 94 (62.7%) were underweight, although these parameters did not differentiate according to thrombotic risk.

The hematological parameters assessed reported a high frequency of anemia (94.7%), although they did not differentiate according to thrombotic risk. This is thought to be the result of frequent hemolysis due to the premature destruction of sickle‐shaped red blood cells, which reduces their lifespan [44]. Many studies have made the same report, among others Tuono et al., whose studies each reported a high frequency of anemia in sickle cell patients, explained by the high frequency of hemolysis due to the abnormal structure of hemoglobin [41, 45]. Furthermore, of the reported anemias, 40.0% were microcytic, 34.7% hypochromic, and 94.7% regenerative although these parameters do not differentiate according to thrombotic risk. The above is the result of a blunted response to erythropoietin secretion associated with the disease. This may be due to a right‐shifted hemoglobin dissociation curve observed in SCD [46].

Furthermore, a significant frequency of leukocytosis (82.7%) in the study population was reported although not differentiating according to thrombotic risk; leukocytosis reported during SCD would reflect the constant absence of inflammation with the resulting release of cytokines, ultimately leading to an increase in leukocyte production in the bone marrow [47]. Rasaki et al. [48] also report cases of leukocytosis (80.8%) during SCD. This has also been reported by other authors [49, 50]. Additionally, 96 (64.0%) patients presented cases of thrombocytosis; thrombocytosis in SCD is variably attributed to anemia induced by increased erythropoietin secretion which has similar homology to thrombopoietin with thrombopoiesis and decreased platelet accumulation in the splenic circulation due to functional and/or structural asplenia characteristic of major sickle cell syndrome [51, 52]. Several authors also report cases of thrombocytosis during SCD [49, 53].

In this study, patients with thrombotic risk had an elevation of inflammatory parameters characterized by an elevation of CRP (100% of patients with the risk versus none in the group without it) and a significantly higher median IL‐6 level in the group of patients with thrombotic risk (50.1 [28.7–76.6] [pg/mL]) compared to those without it (32.3 [14.1–44.4] pg/mL) (*p* = 0.001). Indeed, there is a close relationship between the latter and hemostatic disorders in general and coagulation inhibitors in particular. C‐reactive protein, an acute‐phase reactant, is produced by the liver in response to pro‐inflammatory cytokines (such as TNF‐α, IL‐1β, IL‐6, and IL‐8) and is released into the bloodstream early in the inflammatory process [28, 54]. Inflammation is known to activate coagulation pathways, particularly in SCD, where vaso‐occlusion is characterized by the adhesion of leukocytes to endothelial cells, endothelial dysfunction, and altered nitric oxide metabolism, leading to ischemia–reperfusion injury [36, 55]. Furthermore, abnormal interactions between erythrocytes and the endothelium trigger microvascular occlusions, and a significant correlation has been observed between the clinical severity of SCD and the degree of red blood cell adhesion; exacerbated by the absence of HU therapy, the thrombotic risk was notably higher in patients not receiving this treatment. Physiologically, HU is known to reduce the adhesion of reticulocytes and neutrophils to the vascular endothelium and to downregulate tissue factor expression. Without this modulation, increased endothelial activation and enhanced cell–cell interactions promote thrombin generation [36]. Thus, this study reports a general decrease in physiological coagulation inhibitors in the general population characterized by a significant decrease in protein C in patients with thrombotic risk versus those without, with respective proportions of 71.6% and 50% (*p* = 0.001). The decrease in the latter following SCD was identified as a predictive factor for thrombotic risk during SCD (OR = 3.62 [1.74; 7.52]; *p* < 0.001). This would be the result of a part of persistent systemic inflammation during SCD, which is the origin of an increased consumption of protein C [56], thus reducing its concentration in the plasma; however, products of intravascular hyperhemolysis in sickle cell patients with, among other things, abundant‐free hemoglobin in the circulation, would contribute to reducing the bioavailability of protein C; and in sickle cell patients, the often‐damaged vascular endothelium would therefore alter its capacity to activate protein C adequately due to the ineffectiveness of its membrane receptor, in particular thrombomodulin, in an inflammatory environment [57, 58]. These results are similar to those of Aysun et al. who reported an average of 80.2 ± 20.2% in SS sickle cell patients [59].

However, patients with thrombotic risk presented a significant decrease in protein S (76.6% versus 50% [*p* < 0.001]); the decrease in the latter was identified as a predictive factor of thrombotic risk during SCD (aOR = 1.5 [1.1; 5.2]; *p* = 0.04). This could be explained among other things by the liver, which is the main site of synthesis of protein S, which, due to iron overload, damage due to hemolysis and chronic inflammation; excess iron is often stored in the liver leading to hepatic iron overload (hemosiderosis), which is toxic for liver cells; and the liver then functions suboptimally in sickle cell patients, thus limiting the production of protein S [60, 61]. These observations were also made by Nefissi et al. [29]. Moreover, all patients with thrombotic risk had a decrease in antithrombin with a significantly low median rate (79.0% [77.0–92.0]) compared to those without risk (94.0% [84.0–102]) (*p* < 0.001). This could be explained by the fact that in SCD, chronic inflammation (intrinsic to the disease) promotes a continuous state of coagulation; this activation leads to an increased consumption of antithrombin in an attempt to regulate this prothrombotic state; and this phenomenon is often described as compensated intravascular coagulation [62]. Also, frequent damage to endothelial cells caused by sickle red blood cells leads to a release of procoagulant factors and decreases the production of molecules that stabilize antithrombin levels, which contributes to lowering the levels of this protein [63]. These observations were also made by Olusola et al. [64].

In this study, the role of inflammation in the hypercoagulable state of SCD was further explored through correlation analyses between IL‐6 and natural anticoagulants. Our findings revealed a negative trend between IL‐6 and protein C levels (*r* = −0.16; *p* = 0.051). Although this correlation reached near‐statistical significance, it suggests that systemic inflammation may contribute to the consumption or downregulation of the protein C pathway [65]. This is consistent with the known pathophysiology of SCD, where inflammatory cytokines, such as IL‐6, can suppress the synthesis of protein C or promote its consumption through endothelial activation [66, 67]. The fact that other anticoagulants, such as protein S and antithrombin, did not show significant correlations (*p* > 0.05) might indicate that the protein C system is particularly sensitive to the inflammatory stress in our specific patient cohort. These results, while preliminary, underscore the importance of considering the “inflammation‐hemostasis” axis to better understand thrombotic risk in these patients.

### 4.1. Study Limitation

This study provides current information on thrombotic risks, consequences of inflammation, and homeostasis disorders of physiological coagulation inhibitors in SCD. However, a multicenter and case–control study would have allowed better elucidation of the hypotheses put forward and the final conclusions obtained. Also, investigating liver function parameters might have provided further insights into these associations.

## 5. Conclusion

This study aimed to evaluate physiological coagulation inhibitors and their association with inflammation and to deduce the thrombotic risk in sickle cell patients. It reported a high frequency of thrombotic risk (65.34%). It also reported significant inflammation characterized by an elevation of inflammatory parameters, notably an elevation of CRP (100% of patients at risk versus none in the group without it) and a significantly higher median IL‐6 level in the group of patients at thrombotic risk (50.1 [28.7–76.6] [pg/mL]) compared to those without it (32.3 [14.1–44.4] pg/mL) (*p* = 0.001). Furthermore, the general population showed a decrease in physiological coagulation inhibitors, notably a decrease in protein C, characterized by a significant decrease in the latter in patients at thrombotic risk versus those without, with respective proportions of 71.6% and 50% (*p* = 0.001). Also, patients showed a significantly greater decrease in protein S in the group of patients at thrombotic risk; all patients at thrombotic risk had a decrease in antithrombin with a significantly low median level (79.0% [77.0–92.0]) compared to those without risk (94.0% [84.0–102]) (*p* < 0.001). These observations highlight the need for systematic exploration of these markers for the prevention of cardiovascular disease in this group of patients.

## Author Contributions

Paule Rita Ngamgwe Tchouanmo carried out the data collection and analysis of the biological samples and drafted the manuscript. Romaric De Manfouo Tuono, Ingrid Worti Sulem Yong, and Maryline Seuko Njopwouo interpreted the data and contributed to the drafting of the manuscript. Josué Louokdom Simo and Claude Tagny Tayou contributed to the drafting of the manuscript and supervised the work. Romaric De Manfouo Tuono had full access to all of the data in this study and took complete responsibility for the integrity of the data and the accuracy of the data analysis. Claude Tagny Tayou reviewed the paper.

## Funding

No funding was received for this manuscript.

## Disclosure

All authors read and approved the final version of the manuscript. A preprint version of this study is available online [22].

## Conflicts of Interest

The authors declare no conflicts of interest.

## Data Availability

The data that support the findings of this study are available from the corresponding author upon reasonable request.
